# Evaluation of the mechanism of action of *Bacillus* spp. to manage *Meloidogyne incognita* with split root assay, RT-qPCR and qPCR

**DOI:** 10.3389/fpls.2022.1079109

**Published:** 2023-01-20

**Authors:** Kaitlin M. Gattoni, Sang Wook Park, Kathy S. Lawrence

**Affiliations:** Department of Entomology and Plant Pathology, Auburn University, Auburn, AL, United States

**Keywords:** *Bacillus* spp., cotton, *Meloidogyne incognita*, *B. amyloliquefaciens*, *B. firmus* I-1582, *B. mojavensis*, split root experiments

## Abstract

The goal of this research is to determine the mechanism of action of two *Bacillus* spp. that can manage *Meloidogyne incognita* population density in cotton. The overall objectives are 1) determine the efficacy and direct antagonistic capabilities of the *Bacillus* spp. and 2) determine the systemic capabilities of the *Bacillus* spp. The greenhouse *in planta* assay indicated *B. amyloliquefaciens* QST713 and *B. firmus* I-1582 could manage *M. incognita* similarly to the chemical standard fluopyram. An *in vitro* assay determined that *B. firmus* I-1582 and its extracted metabolites were able to directly manage *M. incognita* second stage juveniles by increasing mortality rate above 75%. A split root assay, used to determine systemic capabilities of the bacteria, indicated *B. amyloliquefaciens* QST713 and *B. firmus* I-1582 could indirectly decrease the nematode population density. Another species, *B. mojavensis* strain 2, also demonstrated systemic capabilities but was not a successful biological control agent because it supported a high population density in greenhouse *in planta* assay and in the split root assay. A RT-qPCR assay was used to confirm any systemic activity observed in the split root assay. At 24 hours both *B. amyloliquefaciens* QST713 and *B. firmus* I-1582 upregulated one gene involved in the initial stages of JA synthesis pathway but not another gene involved in the later stages of JA synthesis. These results point to a JA intermediate molecule, most likely OPDA, stimulated by the bacteria rather than JA in a short-term systemic response. After 1 week, the *Bacillus* spp. stimulated a SA-responsive defense related gene. The long-term systemic response to the *Bacillus* spp. indicates salicylic acid also plays a role in defense conferred by these bacteria. The final assay was a qPCR to determine the concentration of the bacteria on the cotton roots after 24 days. *Bacillus amyloliquefaciens* QST713 and *B. firmus* I-43 1582 were able to colonize the root successfully, with the concentration after 24 days not significantly differing from the concentration at inoculation. This study identifies two bacteria that work *via* systemic resistance and will help aid in implementing these species in an integrated management system.

## Introduction

*Meloidogyne incognita* (Kofoid and White, 1919) Chitwood, the southern root-knot nematode, is an endoparasitic nematode that feeds on hundreds of susceptible plant hosts. The nematode is a major yield-limiting pathogen of cotton, *Gossypium hirsutum* L. *Meloidogyne incognita* caused an estimated 628,600 bale yield reduction in 2017 and a 483,300 bale yield reduction in 2018 across the United States (Lawrence et al., 2018; Lawrence et al., 2019). There are various management options available, the most common being chemical nematicides. However, chemical nematicides can be harmful to the environment and very costly if a nematode problem is widespread in a field (Starr et al., 2007). Crop rotation can be an effective management strategy, but it is not always feasible due to the different expensive equipment needed to harvest and maintain different crops (Kirkpatrick and Sasser, 1984). Biological control has been explored for its ability to manage *M. incognita* in cotton due to the low manufacturing cost and expected environmentally friendliness (Bale et al., 2008).

Biological control agents are one or more organisms, typically fungi or bacteria, which reduce the severity or incidence of a plant disease (Cook and Baker, 1983). There are two proposed mechanisms of action for biological control agents; direct or indirect antagonism. Direct antagonism often involves the release of metabolites, predation or competition (Kerry, 2000). The most common of which is the release of metabolites and predation; competition is very rarely seen or used as biological control strategy. *Pseudomonas flourescens* CHA0, for example, releases two metabolites, 2,4-diacetylphloroglucinol and pyoluteorin, that can significantly decrease *M. javanica* population density (Hamid et al., 2003). A biological control agent that works *via* predation is *Pastueria penetrans* which infects *Meloidogyne* spp., feeds and reproduces within the nematode (Bhuiyan et al., 2018). Determining the direct antagonistic abilities of biological control agents to manage nematodes is often fairly simple. Bacteria and fungi can be screened by an *in vitro* assay that will quickly determine any direct antagonism of nematodes (Xiang et al., 2017). The other biological control strategy, indirect resistance, is not as easily observed and organisms that work by this strategy can be overlooked. Indirect antagonism occurs by systemic resistance, which encompasses induced systemic resistance and systemic acquired resistance. Induced systemic resistance (ISR) is the enhanced disease resistance stimulated typically *via* jasmonic acid (JA) that is produced upon plants’ encounter with plant growth promoting rhizobacteria (PGPR). Plants induce JA upon infections of pathogens, PGPR or herbivores (Seo et al., 1995). Following JA biosynthesis, bioactive JA-Ile binds a SCF ubiquitin E3 ligase, CORONATINE INSENSITIVE1 (COI1), to recruit and ubiquitinate JASMONATE JIM-domain (JAZ) proteins, transcription inhibitors of JA-responsive defense genes (Sheard et al., 2010; Hickman et al., 2017). JA-responsive defense genes include *PDF1.2*, *HEL*, *Thi2.1* and *Chib*, which encode defense proteins, plant defensin 1.2, hevien-like protein, thionin and basic chitinase, respectively (Penninckx et al., 1996; Thomma et al., 1998; Pierterse and van Loon, 1999; Bertini et al., 2012). Systemic acquired resistance (SAR) is a state of heightened defense that is activated through salicylic acid (SA) signaling. SA signaling is stimulated by the recognition of pathogens on the cell surface (Macho and Zipfel, 2015). SA is well known to activate a NONEXPRESSOR OF PATHOGENESIS-RELATED PROTEIN 1 (NPR1) which are transcriptional regulators of SA-responsive defense genes (Maier et al., 2010; Fu et al., 2012; Wu et al., 2012). Both defense hormones and systemic resistance pathways play a role in plant defense against nematodes. Exogenous application of methyl jasmonate, which creates a positive feedback loop for JA, was able to decrease *M. incognita* population density (Fujimoto et al., 2011). When JA biosynthetic pathway was silenced in rice and tomatoes, plant defenses against *Meloidogyne* spp. were unsuccessful (Nahar et al., 2011; Martinez-Medina et al., 2017). SA is known to be heavily involved in *Mi-1* resistance, the main form of plant resistance to *M. incognita* (Branch et al., 2004). As well as being involved in nematode defense, both of these hormones can be stimulated by PGPR. There are more examples of JA stimulation by PGPRs, including strains of *B. subtilis*, *B. cereus*, *B. pumilus*, and *B. amyloliquefaciens* and species of *Pseudomonas*, than there are examples of SA stimulation (Yan et al., 2002; Murphy et al., 2003; Beneduzi et al., 2012; Garcia-Gutierrez et al., 2013) However, there are a few instances where SA is upregulated by PGPR, including *B. amyloliquefaciens* LJ02, *B. amyloliquefaciens* strain MBI600, *Pseudomonas aurofaciens* and *P. corrugata* (Chen et al., 1999; Li et al., 2015; Beris et al., 2018). Stimulation of SA or JA by PGPRs can be a successful indirect management strategy of nematodes. Many *Bacillus* spp. are effective in biological control against different pathogens. Some *Bacillus* spp. use secondary metabolites and anti-microbial properties as a form of direct antagonism to reduce disease and pests. An example of this is the synthesis of a bacillomycin D by *B. subtilis* which can inhibit spore germination and sporulation of *Aspergillus* spp. (Gong et al., 2014). Similarly, a strain of B. *amyloliquefaciens* caused abnormal germination of various fungi, including *Fusarium* and *Aspergillus* spp., because it produces secondary metabolite such as iturins-like and fengycin-like peptides (Benitez et al., 2010). There are many other examples of different *Bacillus* spp., mainly strains of *B. subtilis*, which reduce bacterial or fungal diseases by release of metabolites and exhibiting anti-microbial properties. Fewer examples of *Bacillus* spp. that release metabolites that impact nematodes are documented. In one study, *M. incognita* was decreased *in vitro* by secondary metabolites extracted from *B. firmus* (Mendoza et al., 2008). Another study looked at over 600 *Bacillus* strains and demonstrated that 33% of those, from various species, were able to increase the percent mortality of *M. incognita in vitro* (Xiang et al., 2017). This was attributed to direct antagonism against the nematode, which is predicted to be by the release of metabolites. A similar study evaluated *B. cereus* strain S2 *in vitro* and determined it released sphingosine to cause reactive oxygen (ROS) response in *M. incognita* which resulted in cell necrosis and injury in nematodes (Gao et al., 2016). SA and SAR are less frequently seen to be correlated with *Bacillus* spp., however there are some examples of this. Li et al. (2015) found that *B. amyloliquefaciens* LJ02 stimulated SA activity and the activation of PR11 in cucumber. This species was tested previously to be effective in managing *Fusarium oxysporum*, *Botrytis cinerea* and *Alternaria* spp. in cucumber (Li et al., 2015). Another strain, *B. amyloliquefaciens* strain MBI600 from the commercial product Serifel^®^ (BASF SE) was seen to induce salicylic acid to reduce the disease severity of tomato spotted wilt virus in tomatoes (Beris et al., 2018).

While there are fewer examples of SA acid stimulated by *Bacillus* spp. then there are examples of JA stimulation. Few studies, however, look at both hormone pathways when analyzing systemic resistance. Ghahremani et al. (2020), found SA and JA related genes were primed at different times after *M. incognita* inoculation in tomato, but only the SA-related gene was up-regulated at 7 days after *M. incognita* in cucumber. It is likely that both pathways are involved in some form of plant defense response to *M. incognita*, therefore it is important to analyze both pathways that could possibly be stimulated by a *Bacillus* spp. biological control agent.

The goal of integrated pest management (IPM) is to effectively manage diseases and pests in crops using an environmentally sensitive approach. Biological control agents are an integral part of IPM. Development of commercial biological control agents requires extensive knowledge of the bacteria or fungi being considered, including the mechanism of action and any interactions with the pest. The goal of this study is to determine the mechanism of action, direct or indirect, of *Bacillus* spp. to help successfully implement them in an IPM setting that also involves cultural control, resistant varieties, and limited use of chemical nematicides. The objectives of this research are to determine the efficacy of select *Bacillus* spp. and their direct effect on *M. incognita* and to determine the systemic capabilities of the *Bacillus* spp. and potentially explain the mechanism of action of the *Bacillus* spp.

## Material and methods

### Nematode inoculum preparations

*Meloidogyne incognita* race 3 was used in the greenhouse *in planta* assay, the *in vitro* assay, the split root assay and the RT-qPCR. Stock pots of the nematode were grown on corn, Mycogen 2H723 (Dow AgroScience, Indianapolis, IN), in 500 cm^3^ polystyrene pots in the greenhouses at the Plant Science Research Center (PSRC) in Auburn, AL. Eggs were extracted by placing the corn roots in a 0.625% NaOCl solution and shaking them for 4 minutes at 1 g-force on a Barnstead Lab Line Max Q 5000 E Class shaker (Conquer Scientific, San Diego, CA). The roots were rinsed with tap water and the eggs were collected on a 25-μm pore sieve. The collected eggs were washed into 50mL centrifuge tubes and processed by sucrose centrifugation at 427 g-forces for 1 minute (Jenkins, 1964). The eggs were recollected on a 25-μm pore sieve. For the greenhouse *in planta* assay and the split root assay, the nematodes were quantified using an inverted TS100 Nikon microscope at 40x magnification. The nematode eggs were standardized to 5,000 eggs per mL. For the *in vitro* assay and RT-qPCR, the eggs were hatched to second stage juveniles (J2) using a modified Baermann funnel (Castillo et al., 2013). The modified Baermann funnel was placed on a slide warmer (Model 77; Marshall Scientific, Brentwood, NH) set to 30°C and left to incubate for 5 to 10 days, dependent on outside temperature and time of the year (Xiang and Lawrence, 2016). The J2s in the Baermann funnel were washed onto a 25-μm pore sieve and collected in a beaker using minimal water. The number of J2s was quantified under an inverted TS100 Nikon microscope at 40x magnification. For the *in vitro* assay the number of J2s was standardized to approximately 30 J2s per 10 µL of water. For the RT-qPCR the number of J2s was standardized to 1,000 juveniles per 1 mL of water.

### Bacterial inoculum preparations

Five *Bacillus* spp. were used in all the assays. Three species, *B. mojavensis* strain 3, *B. velenzensis* strain 2, and *B. pumilis* GB 34 were originally isolated, identified and stored by Dr. J. W. Kloepper at Auburn University, Auburn, AL. Two species, *B. firmus* I-1582 and *B. amyloliquefaciens* QST713, are the active ingredients of Bayer CropScience products, VOTiVO^®^ and Serenade^®^, respectively. The *Bacillus* spp. were stored in 30% glycerol at -80°C. Prior to utilization, the *Bacillus* spp. were transferred to tryptic soy agar (VWR, Radnor, PA) plates and incubated at 35°C for 24 hours. The vegetative cells were washed into beakers and standardized. For the greenhouse *in planta* assay, *in vitro* assay, split root assay and RT-qPCR, the bacteria were standardized to 1 x 10^6^ CFU/mL. For the qPCR, the bacteria were standardized to 1 x10^8^ CFU/mL.

### Greenhouse in planta assay

Soil used in all experiments was a Kalmia loamy sand (80% sand, 10% silt, and 10% clay) obtained from the Plant Breeding Unit located at the E.V Smith Research Center near Tallassee, AL. This soil was brought to the PSRC and pasteurized at 88°C for 12 hours then cooled for 24 hours before the pasteurization was repeated. Four cotton seed (Phytogen 333 WRF) were planted in 500 cm^3^ polystyrene pots filled with 2:1 pasteurized soil to sand mixed with fertilizer and lime as recommended by the Auburn University Soil Lab. The seeds were inoculated at planting with *M. incognita* and a control of 1 mL of water per seed, 1 mL of each of the *Bacillus* spp., or the chemical control of fluopyram (Velum, Bayer CropScience) at a rate of 0.5 µL followed by 1 mL of water per seed. Natural light in the greenhouse at PSRC was supplemented with light from 1,000-watt halide bulbs producing 110,000 lumens to provide 14 hours of light. Temperatures ranged from 22°C to 34°C and tests were watered twice a day. Thirty days after inoculation, plant data [plant height (cm), shoot fresh weight, (g) and root fresh weight (g)] were measured. Biomass (g) was determined by adding the root fresh weight to the shoot fresh weight. The nematodes were extracted from the roots of the cotton plant as previously described. Nematode eggs were quantified under the inverted TS100 Nikon microscope at 40x magnification. This test was a randomized complete block design (RCBD) with 5 replicates of each treatment per assay and the entire experiment was repeated two times for a total of 70 experimental units.

### *In vitro* assay

The *in vitro* assay measured the direct response of the J2 to each bacterial isolate. Bacteria, grown as described above, were incubated for 6 days, after which they were carefully washed into 2 separate 1.5 µL microcentrifuge tubes. The metabolites were extracted from one of the tubes by rotating between a hot water bath and ice bath for 15 minutes. The tubes were then centrifuged for 1 minute following the methodology of Apotroaie-Constantin et al. (2008). The supernatant containing the metabolites was collected after centrifugation. Following metabolite extraction, 10 µL of J2s along with 90 µL of a water control, the extracted metabolites, or the bacterial inoculum was added to each well of a 96 well plate. The fluopyram chemical control was not utilized as it is an opaque white liquid that made determining mortality of nematode under microscopy difficult. At 0 hours and 48 hours the number of live and dead nematodes was quantified using stimulation with sodium hydroxide Xiang and Lawrence (2016), under the inverted TS100 Nikon microscope at 40x magnification. The percent mortality was calculated [(live J2s at 0 hours – live J2s at 48 hours)/live J2s at 0 hours] x 100. The treatments were replicated 5 times per assay and the assays were repeated three times for a total of 90 experimental units.

### Split root assay

**Figure 1:**
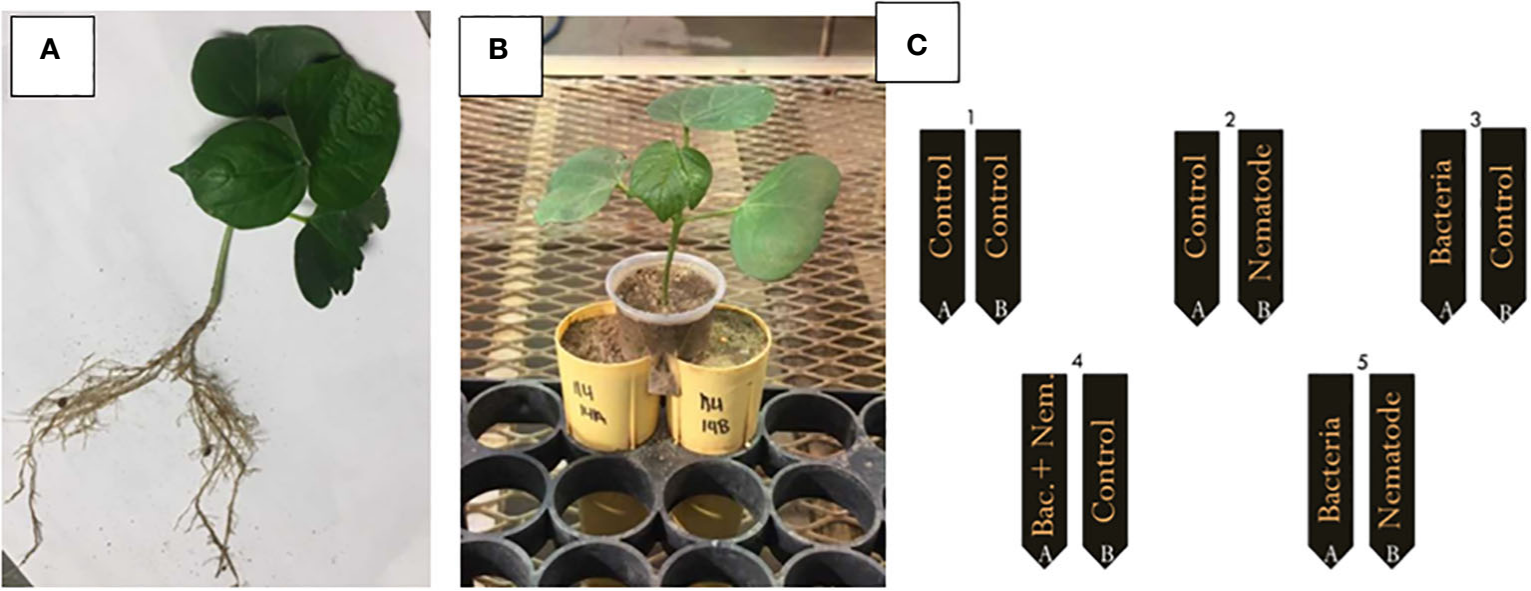
Example of a split root system; **(A)** two equivalent cotton root systems before planting in the split root set up, **(B)** after planting the cotton in the split root set up, and **(C)** inoculation pattern for the split root assay with 1) a control on root half A and B (control), 2) bacteria or fluopyram inoculated on root half A and control on root half B (bacteria control), 3) control on root half A and *Meloidogyne incognita* eggs inoculated on root half B (nematode control), 4) bacteria or fluopyram and *M. incognita* eggs inoculated on root half A and control on root half B, and 5) bacteria or fluopyram inoculated on root half A and *M. incognita* eggs inoculated on root half.

Cotton seeds (Phytogen 333 WRF) were germinated in germination paper on a slide warmer for 4-6 days or until a small taproot had formed. The roots were cut horizontally from the root tip with a sterile razor blade approximately 1 mm from the end. The seedlings were planted in sand supplemented with fertilizer at a rate of four seedlings per pot. The plants were allowed to grow in the greenhouse for 1-2 weeks or until two equivalent root halves were produced (**Figure 1A**). The cotton plants were gently excavated from the soil and the roots were washed very carefully with tap water to remove excess sand. The two root halves of each cotton plant were planted in two 150 cm^3^ conetainers (Stuewe & Sons Inc.; Tangent, Oregon) positioned immediately next to each other filled with 2:1 sand to soil with fertilizer and lime as recommended by the Auburn University Soil Lab. The shoots were positioned in small plastic cups with the bottoms cut off that were positioned equally over the two conetainers containing the two root halves (**Figure 1B**). The root halves were inoculated with the bacteria, nematodes and fluopyram two days after the cotton seedling was transplanted in the split root system. For each bacterial or chemical treatment there were five distinct split root inoculation patterns: 1) a control with no inoculation on either root half (control), 2) bacteria or fluopyram inoculated on root half A and no inoculation on root half B (bacteria control), 3) no inoculation on root half A and *M. incognita* eggs inoculated on root half B (nematode control), 4) bacteria or fluopyram and *M. incognita* eggs inoculated on root half A and no inoculation on root half B, and 5) bacteria or fluopyram inoculated on root half A and *M. incognita* eggs inoculated on root half B (**Figure 1C**; Martinez-Medina et al., 2017). Thirty days after inoculation, the plant parameters were measured as previously described and the nematode eggs were quantified. The split root test was designed in a RCBD. The patterns for each treatment were replicated 5 times per assay and the assay was repeated 3 times for a total of 900 experiment units.

### RT-qPCR

Quantitative real time PCR was used to determine the transcript level of genes related to JA expression (*GhLOX, GhOPR3*; Zebelo et al., 2016) and SA activity (*β-1,3-glucanase*; Zhang et al., 2011). Cotton seeds, as described previously, were planted in containers filled with 2:1 sand to soil with fertilizer and lime as previously described. The cotton was grown in the greenhouse for 2 to 3 weeks, or until the second true leaf stage. Once the cotton plants reached this growth stage the cotton plants were inoculated with *B. amyloliquefaciens* QST713, *B. firmus* I-1582, *M. incognita* J2s, or left untreated as a control. At 0 hours (h), 1h, 24h, and 1 week, approximately 2 grams of roots were placed in a 1.5 mL microcentrifuge tube and immediately frozen in liquid nitrogen. The samples were kept in -80°C until ready for use. The samples were ground in liquid nitrogen into a fine powder using a mortar and pestle. RNA was extracted using the Spectrum™ Plant Total RNA Kit (Sigma Aldrich, St. Louis, MO, USA) according to manufacturer instructions. Concentration and purity of the RNA was determined using the NanoDrop™ Spectrophotometer ND-2000 (*A_260_*/*A_280_* > 1.8 and *A_260_*/*A_230_* > 2.0; Thermo Scientific, Wilmington, USA). RT reactions were carried out using the GoScript™ reverse transcription system Kit (Promega, Madison, WI, USA), and qPCR was performed with the PerfeCTA^®^ SYBR^®^ Green Fastmix^®^ qPCR Master Mix (Qunita Biosciences, Inc, Gaithersburg, MD, USA) in a CFX96 RealTime System (Bio-Rad) cycled 40 times using gene specific primer sets (**Table 1**; Invitrogen, ThermoFisher Scientific; Waltham, MA). The annealing temperatures for the primer pairs were 60°C. Relative RNA levels were calibrated and normalized with a housekeeping gene, *HISTONE H3*. Relative fold change was calculated by normalizing the average threshold cycles (Ct) of target genes to that of *H3* as 2^−ΔCT^, where –ΔCT = (C*_t’gene_*-C*_t’H3_*) (Livak and Schmittgen, 2001). There were 6 biological replicates per treatment, for a total of 96 experimental units, and 3 technical replicates for each biological sample.

**Table 1:** Primers for *GhLOX1*, *GhOPR3* and *β-1,3, glucanase*.

Gene	Forward (5’ to 3’)	Reverse (5’ to 3’)	Reference
Histone (H3)	GAAGCCTCATCGATACCGT	CTACCACTACCATCATGGC	Zebelo et al., 2016
*GhLOX1*	GCCAAGGAGAGCTTCAAGAAT	TAGGGGTACTTGGCAGAACCT	Zebelo et al., 2016
*GhOPR3*	ATGTGACGCAACCTCGTTATC	CCGCCACTACACATGAAAGTT	Zebelo et al., 2016
*β-1,3, glucanase*	AATGCGCTCTATGATCCG	GATGATTTATCAATAGCAGCG	Zhang et al., 2011

### qPCR for bacterial concentration

The protocol to determine the concentration of the bacteria on the roots was adapted for cotton from Mendis et al. (2018). Cotton seeds were planted in conetainers filled with 2:1 sand to soil with fertilizer and lime as previously described. The plants were inoculated at planting with *B. firmus* I-1582 and *B. amyloliquefaciens* QST713. After 24 days of growth, the roots were gently shaken to remove most excess soil retaining the rhizosphere soil on the roots. An amount of 1.5 g of sampled roots and rhizospheric soil was added to a 7mL plastic vial (BioSpec Products Inc, Bartlesville, OK) filled with approximately 1.75 g of 2 mm Zirconia beads (BioSpec Products Inc, Bartlesville, OK). A volume of 2 mL of sterile water was added to each vial. The vials were beadbeated in a Mini-BeadBeater-96 (BioSpec Products Inc, Bartlesville, OK) at 2,400 oscillations/min for 5 minutes. The vials were then centrifuged at 427 g-forces for 1 second. A volume of 200 µL was taken from the supernatant and added to the ZR BashingBead Lysis Tube from the ZR Soil Microbe DNA miniprep kit (Zymo Research Corporation, Irvine, CA). The DNA was extracted according to the manufacturer’s instructions.

qPCR was carried out on a CFX96 RealTime System (Bio-Rad) using primers and TaqMan^®^ probes developed by Mendis et al. (2018). qPCR was done with PerfeCTa Multiplex qPCR ToughMix Low ROX (Quantabio, Beverly, MA), and the annealing temperatures were 95° C for 39 cycles. There were 15 biological samples per treatment, for a total of 30 experimental units, and 3 technical replicates of each biological sample.

DNA was extracted from samples with a known concentration of bacteria to create a standard curve used to calculate the concentration of the experimental samples based on the Cq values. A serial dilution of bacteria, 10^2^ to 10^9^ cfu/mL, was created. A volume of 1 mL of each serial dilution was added to 1.5 g of cotton roots in 7mL plastic vial filled with approximately 1.75 g of 2 mm Zirconia beads, as previously described. A volume of 1 mL of sterile water was added to this before the samples were processed and the DNA was extracted as previously described. qPCR was performed as previously described. The Cq value was plotted against the log value for the serial dilution to get a standard curve. The experimental concentrations were calculated with the slope of the standard curve and the experimental Cq values.

### Statistical analysis

Data collected from the greenhouse *in planta* assay, *in vitro* assay, split root assay and qPCR for bacterial concentration were analyzed in SAS 9.4 (SAS Institute, Cary, NC) using the Glimmix procedure with means separated by use of the Tukey-Kramer method with a significance level of *P* ≤ 0.05 or *P* ≤ 0.10. For the RT-qPCR, the data were statistically analyzed in SAS 9.4 (SAS Institute, Cary, NC) by two-way ANOVA with a level of significance of *P* ≤ 0.001, *P* ≤ 0.01, or *P* ≤ 0.05.

## Results

### Greenhouse in planta assay

In the greenhouse *in planta* assay, two of the five *Bacillus* spp. reduced *M. incognita* population density. *Bacillus firmus* I-1582 and *B. amyloliquefaciens* QST713, decreased the nematode eggs per gram of root compared to the control, similarly to fluopyram (*P*≤ 0.05; **Table 2**). The number of nematode eggs per gram of root was also decreased by the chemical standard of fluopyram compared to the control. The plant parameters, including plant height, shoot fresh weight, root fresh weight and biomass, did not differ between any of the treatments 30 days after inoculation.

**Table 2:** Greenhouse *in planta* test to evaluate five *Bacillus* spp. as biological control agents of *Meloidogyne incognita* on cotton as measured by plant height, shoot fresh weight (SFW), root fresh weight (RFW), biomass and *M. incognita* eggs/gram of root 30 days after inoculation^y^.

Treatment	Plant height (cm)	SFW (g)	RFW (g)	Biomass (SFW+RFW)	*M. incognita* eggs/ g of root
Control	15.26	2.79	1.52	4.31	4561 a^z^
Fluopyram	15.40	2.96	1.54	4.50	40 c
*B. firmus* I-1582	14.16	2.52	1.19	3.71	1135 bc
*B. amyloliquefaciens* QST713	15.26	3.24	1.59	4.83	951 bc
*B. pumilus* GB34	15.29	3.01	1.35	4.36	2520 abc
*B. velenzensis* strain 2	14.75	2.66	1.34	4.00	1930 abc
*B. mojavensis* strain 3	14.49	2.82	1.36	4.18	3853 ab

y Randomized complete block design (RCBD) with five replicates of each treatment per assay and he tests was repeated twice.

z Data were statistically analyzed in SAS 9.4 using the Glimmix procedure with means separated utilizing Tukey-Kramer’s method P ≤ 0.05. Values in the same column followed by different letters are significantly different.

### *In vitro* assay

The percent mortality ranged from 6.1% to 78.7% with the lowest mortality rate occurring in the water control (**Table 3**). *Bacillus firmus* I-1582 and the *B. firmus* I-1582 metabolites increased percent mortality significantly, by 77.8% and 78.7% respectively, compared to the water control (*P*≤ 0.05; **Table 3**). The *B. amyloliquefaciens* QST713 metabolites also increased percent mortality, by 62.2%, compared to the control; however, the *B. amyloliquefaciens* QST713 intact bacteria did not, only increasing percent mortality by 33.8% (*P*≤ 0.05; **Table 3**).

**Table 3:** *In vitro* assay to determine the percent mortality of *Meloidogyne incognita* J2s 48 hours after exposure to five *Bacillus* spp.

Treatment	Percent of J2 mortality^x^
Water control^y^	6.1 c^z^
*B. firmus* I-1582	77.8 a
*B. firmus* I-1582 metabolites	78.7 a
*B. amyloliquefaciens* QST713	33.8 bc
*B. amyloliquefaciens* QST713 metabolites	62.2 ab
*B. pumilus* GB34	38.6 bc
*B. pumilus* GB34 metabolites	35.7 bc
*B. velenzensis* strain 2	24.5 c
*B. velenzensis* strain 2metabolites	30.5 c
*B. mojavensis* strain 3	24.7 c
*B. mojavensis* strain 3 metabolites	20.6 c

x Assays were performed in 96 well plates.

y Percent mortality calculated using this formula: ([(live J2s at 0 hours – live J2s at 48 hours)/live J2s at 0 hours] x 100).

z All treatments were done in a replicate of 5 per assay and the assay was repeated 3 times. Data were statistically analyzed in SAS 9.4 using the Glimmix procedure with means separated utilizing Tukey-Kramer’s method P ≤ 0.05. Values in the same column followed by different letters are significantly different.

### Split root assay

There was no difference in the plant parameters between the split root treatments; however, there was a difference in the eggs per gram of root. Fluopyram inoculated concomitantly on the same root half as the nematode decreased *M. incognita* numbers by 94% compared to the nematode control (*P*≤ 0.05; **Table 4**). *Bacillus firmus* I-1582 decreased nematode numbers by 78% when the bacteria and nematode were inoculated concomitantly on the same root half and by 84% when inoculated on the opposite root half, as compared to the nematode control (*P*≤ 0.05; **Table 4**). Similarly, *B. amyloliquefaciens* QST 713 decreased nematode numbers by 68% when the bacteria and nematode were inoculated concomitantly on the same root half and by 86% when inoculated on the opposite root half, as compared to the nematode control (*P*≤ 0.05; **Table 4**). *Bacillus mojavensis* strain 3 decreased nematode numbers by 82% when the bacteria and nematode were inoculated on the opposite root half, as compared to the nematode control (*P*≤ 0.05; **Table 4**). This species did not decrease nematode numbers when inoculated on the same root half as the nematode, having the largest number of nematode eggs of all the *Bacillus* spp. and chemical treatments (*P*≤ 0.05; **Table 4**). This indicates that *B. mojavensis* strain 3 is not a good control option for *M. incognita* and was not used in the RT-qPCR. The two bacteria that exhibited systemic responses, *B. amyloliquefaciens* QST 713 and *B. firmus* I-1582, were analyzed further with RT-qPCR.

**Table 4:** Split root assay to measure the effect of the *Bacillus* spp. on *Meloidogyne incognita* on cotton as measured by plant height, shoot fresh weight (SFW), root fresh weight (RFW), biomass and *M. incognita* eggs/ gram of root 30 days after inoculation^w^.

Treatment^x^	Plant height (cm)	SFW (g)	RFW (g)	Biomass (SFW +RFW)	*M. incognita* eggs/g of root	
Control (A) Control (B)	16.79	3.82	1.44	5.26	NA^y^	
Control (A) *M. incognita* (B)	16.24	3.40	2.14	5.55	21335	a^z^
*B. firmus* I-1582 (A) Control (B)	18.46	4.35	1.70	6.06	NA	
*B. firmus* I-1582 + *M. incognita* (A) Control (B)	17.24	4.76	2.09	6.85	4648	bc
*B. firmus* I-1582 (A) *M. incognita* (B)	15.24	4.35	1.80	6.47	3386	bc
*B. amyloliquefaciens* QST713 (A) Control (B)	16.46	4.13	1.94	6.08	NA	
*B. amyloliquefaciens* QST713 + *M. incognita* (A) Control (B)	15.68	4.03	1.56	5.60	6912	bc
*B. amyloliqeufaciens* QST713 (A) *M. incognita* (B)	16.88	4.10	1.47	5.58	2922	bc
*B. pumilus* GB34 (A) Control (B)	16.91	4.23	1.77	6.16	NA	
*B. pumilus* GB34 + *M. incognita* (A) Control (B)	15.91	3.82	1.98	5.80	11033	abc
*B. pumilus* GB34 (A) *M. incognita* (B)	16.46	3.84	1.62	5.47	12498	ab
*B. velenzensis* strain 2 (A) Control (B)	14.57	3.44	1.96	5.41	NA	
*B. velenzensis* strain 2 + *M. incognita* (A) Control (B)	16.02	3.51	1.38	4.90	15522	ab
*B. velenzensis* strain 2 (A) *M. incognita* (B)	15.68	3.55	1.79	5.34	9104	abc
*B. mojavensis* strain 3 (A) Control (B)	15.68	4.25	1.82	6.07	NA	
*B. mojavensis* strain 3 + *M. incognita* (A) Control (B)	17.02	4.00	1.80	5.80	18904	ab
*B. mojavensis* (A) *M. incognita* (B)	15.62	3.46	1.54	5.00	3848	bc
Fluopyram (A) Control (B)	15.91	3.49	1.69	5.18	NA	
Fluopyram + *M. incognita* (A) Control (B)	14.91	3.39	1.47	5.06	1190	c
Fluopyram (A) *M. incognita* (B)	17.46	3.90	1.74	5.65	10020	abc

^w^Randomized complete block design (RCBD) with five replicates of each treatment per assay and three replicates of the assay was used.

^x^Split root inoculation patterns in **Figure 1**.

^y^NA indicated no nematodes were added.

^z^Data were statistically analyzed in SAS 9.4 using the Glimmix procedure with means separated utilizing Tukey-Kramer’s method P ≤ 0.1. Values in the same column followed by different letters are significantly different.

### RT-qPCR

**Figure 2:**
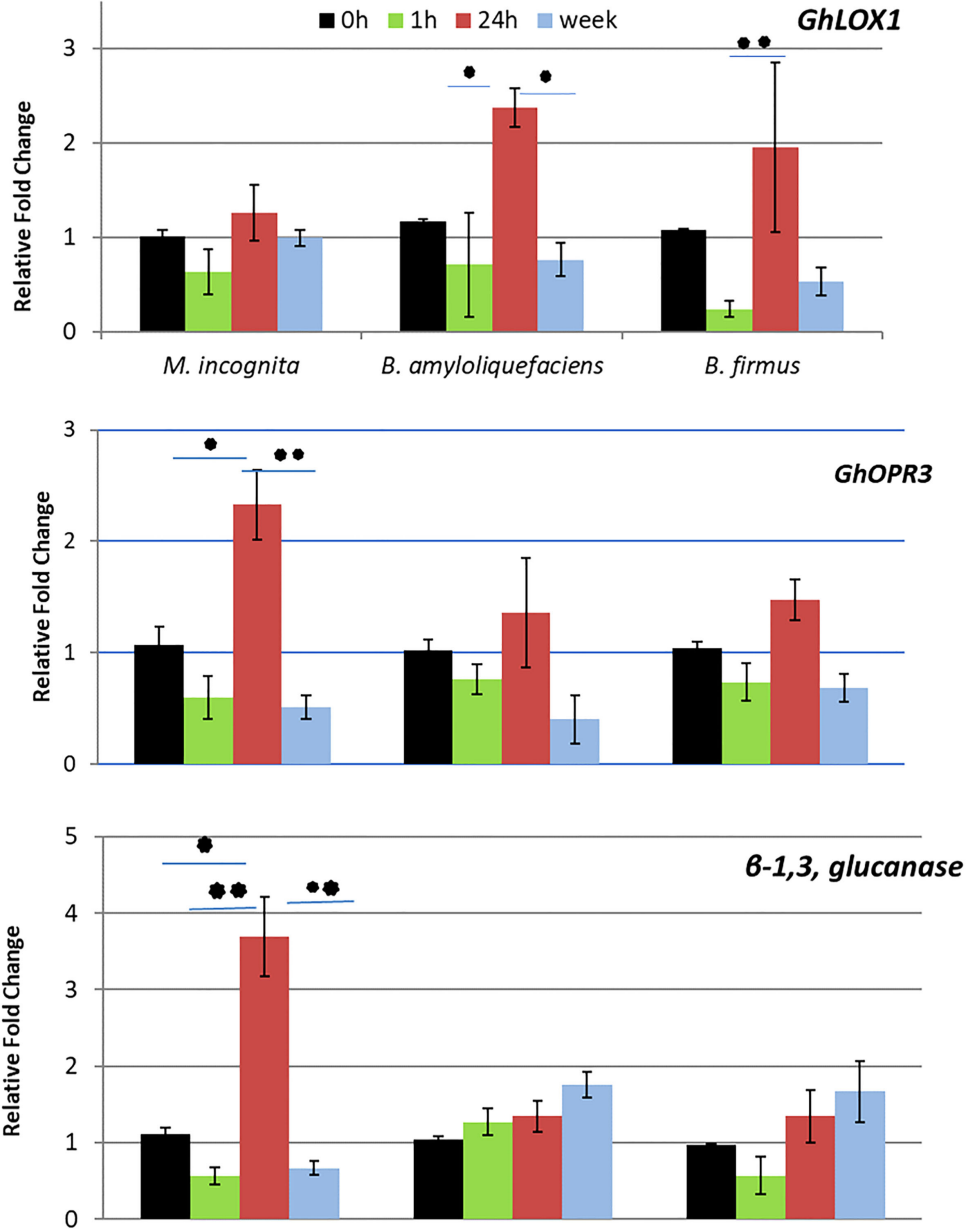
Results from the RT-qPCR depicting the relative fold change of *GhLOX1, GhOPR3, β-1,3, glucanase* in cotton at 0 hours (h), 1h, 24h and 1 week after inoculation of *Meloidogyne incognita* J2s, *Bacillus amyloliquefaciens* QST713 and *B firmus* I-1582. Fold change was calculated 2−ΔΔC T (Livak and Schmittgen, 2001) where the treated samples were compared to a negative control group and normalized with the reference gene histone H3. Asterisks represent significance as determined by a two way ANOVA performed in SAS 9.4 (*P ≤ 0.05; **P ≤ 0.01).

The RT-qPCR was conducted to analyze genes correlated to systemic resistance. *GhLOX1* and *GhOPR3* typically correlate to JA regulation or regulation of an intermediate jasmonate defense molecule. *β-1,3-glucanase* correlates with SA activity. *GhLOX1* expression displayed significant upregulation at 24-hour post *Bacillus* spp. inoculation, whereas *GhOPR3* expression was upregulated at 24 hours after stimulation by the nematode but not the *Bacillus* spp. (**Figure 2**. At 24 hours, *β-1,3-glucanase* was also significantly upregulated by the nematode. At all other time points, the nematode downregulated *β-1,3-glucanase* (**Figure 2**). In contrast, stimulation by the *Bacillus* spp. led to a steady increase of *β-1,3-glucanase* expression. At one week, both *Bacillus* spp. significantly upregulated *β-1,3-glucanase* compared to the nematode.

### qPCR for bacterial concentrations

**Figure 3:**
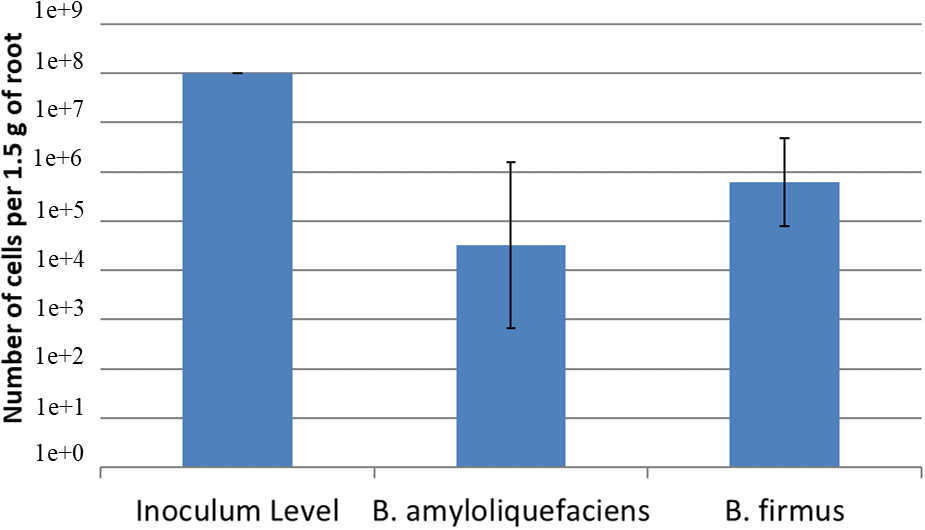
Quantification of *Bacillus amyloliquefaciens* QST713 and *B firmus* I-1582 after 24 days on cotton in the greenhouse using qPCR. Cotton seeds were inoculated with 10^8^ cfu/mL of each of the bacteria at day 0. The concentration was calculated using the standard curves for each bacteria; *B amyloliquefaciens* QST713: y = -1.9421x + 34.227; R^2^ = 09371; *B firmus* QST713 I-1582: y = -2.2036x + 44.362; R^2^ = 08217. The treatments were done in a replicate of 5 per assay and the assay was repeated 3 times. Data were statistically analyzed in SAS 9.4 using the Glimmix procedure with *P ≤* 0.1. The treatments did not significantly differ from each other.

Using a serial dilution of *B. amyloliquefaciens* QST713 and *B. firmus* I-1582, two standard curves were created. The equation for the *B. amyloliquefaciens* QST713 was y = -1.9421x + 34.227; R^2^ = 09371. The equation for the *B. firmus* QST713 I-1582 was y = -2.2036x + 44.362; R^2^ = 08217. The inoculum level of the bacteria at day 1 was 10^8^ cfu/mL of both *B. amyloliquefaciens* QST713 and *B. firmus* I-1582. After 24 days, qPCR determined that concentration for *B. amyloliquefaciens* QST713 was 10^4.52^ cfu/mL and *B. firmus* I-1582 was at a concentration of 10^5.79^ cfu/mL (**Figure 3**). Indicating these bacteria can successfully colonize the plant roots.

## Discussion

Biological control agents that utilize unique mechanisms of action are a good option for management of *M. incognita* and can be readily implemented into an integrated pest management strategy. *Bacillus* spp. are popular bacteria to consider as biological control agents because they can tolerate harsh environments, easily form endospores, effect a wide range of pathogens, and replicate and colonize quickly (Shafi et al., 2017).

Five *Bacillus* spp. were tested in this study for the ability to manage *M. incognita* population density. In a greenhouse *in planta* test, *B. firmus* I-1582 and *B. amyloliquefaciens* QST713 showed potential for decreasing nematode numbers. An *in vitro* assay determined that one of these species*, B. firmus* I-1582 and its metabolites, was able to directly antagonize the nematode. The percent mortality 48 hour after exposure to the extracted metabolites and the percent mortality of the intact bacteria were equivalent, indicating the bacteria can potentially release the extracted metabolites to antagonize the nematode. This is different from a previous study that looked at the percent mortality of Poncho/VOTiVO^®^, a formulated mixture of *B. firmus* I-1582 and the insecticide clothianidin, in the same style of *in vitro* assay and observed only a 24.4% increase in mortality and no significant difference compared to the control (Xiang et al., 2017). Contradictory to this study and in agreement with our study another researcher found that a similar strain of *B. firmus* was able to manage *M. incognita*, the burrowing nematode *Radopholus similis* and the stem nematode *Ditylenchus dipsaci in vitro* (Mendoza et al., 2007). This may indicate that the bacteria’s ability to directly manage nematodes can be impeded when mixed with other products, in this case an insecticide. On the other hand, *B. amyloliquefaciens* QST713 metabolites also increased percent mortality but the intact bacteria did not, indicating the intact bacteria cannot readily release the metabolites that could result in direct antagonism. There have previously been no examples of this specific strain of *B. amyloliquefaciens* managing nematodes *in vitro* or *in planta*. However, another strain of *B. amyloliquefaciens* was seen to reduce galling in tomatoes (Burelle and Dickson., 2003). As well, in an *in vitro* screening four strains of *B. amyloliquefaciens* increased percent mortality by over 50% (Xiang et al., 2014). There are no studies that look at metabolites released by this bacterium and their effect on nematodes, however. Further studies should determine metabolite identification for both species to determine if they can be utilized in a commercial product.

The split root technique is common in determining systemic resistance, pathogen-pathogen interactions and rhizobium formation on legumes (Selim et al., 2014). Split root assays involve observing the reaction of the responder, *M. incognita* in this case, on one root half to the inducer, *Bacillus* spp., on the other root half. Three species showed potential systemic responses in the split root assay. *Bacillus firmus* I-1582 and *B. amyloliquefaciens* QST713 decreased nematode numbers when inoculated on the same root half as the nematode and when inoculated on the opposite root half as compared to the nematode control. The other bacteria that exhibited systemic resistance, *B. mojavensis* strain 3, only decreased nematode numbers when on the opposite root half as the nematode. When this bacterium was in contact with the nematode in both the split root and greenhouse *in planta* assay, the population density of eggs per gram of root was equivalent to the nematode control, indicating this bacterium will not be a good biological control agent. These results from the split root assay are useful for determining systemic capabilities; however, this assay opens questions as to the specific pathway being stimulated by the *Bacillus* spp.

*Meloidogyne incognita* resistant varieties of tomato often use *Mi* resistance, the most common of which is *Mi-1* resistance (Branch et al., 2004; Bhattarai et al., 2007). *Mi-1* resistance relies on SA, one of the major defense hormones that can be stimulated by biological control agents (Bhattarai et al., 2007). The other major defense hormone involved in pathogen defense is JA, which can inhibit SA production and can be inhibited by SA (Pena-Cortes et al., 1993; Uppalapati et al., 2007). This antagonistic relationship between the hormones is typically based on the concentration of each hormone; the higher the concentration of one, the more antagonistic the relationship (Mur et al., 2006). The use of a *Mi-1* resistant plants along with a biological control agent that significantly stimulates JA may result in less protection against *M. incognita* than expected dependent on the interaction between SA and JA (Gutjahr and Paszkowski, 2009; Fujimoto et al., 2011). The source of a major resistant strain of cotton, Auburn 623 RNR, and other sources of resistance in many different varieties of cotton to *M. incognita* have not been identified yet (Kumar et al., 2016). It is possible that, similarly to tomato *Mi-1* resistance, the major form of resistance in cotton involves one of these two major defense hormones.

To confirm the split root assay results and determine the specific pathway stimulated, a RT-qPCR analyzed the expression level of genes correlating to levels of JA, SA, and a possible intermediate defense molecule. *GhLOX1* is involved in initial JA synthesis within the chloroplast, *GhOPR3* is involved with JA synthesis within the peroxisome and *β-1,3-glucanase* is a SA responsive gene (Breithaupt et al., 2001; Zhang et al., 2011; Zebelo et al., 2016). In our RT-qPCR, *GhLOX1* was upregulated by the *Bacillus* spp. but not the nematode at 24 hours after inoculation. In contrast, *GhOPR3* was upregulated by the nematode but not the *Bacillus* spp. at 24 hours. At all other time points, *GhOPR3* and *GhLOX1* were not upregulated indicating a short-term local response by the plant to the stimulants. The upregulation of *GhOPR3* by the nematode at 24 hours is thought to be the plant’s natural response to the nematode. It is known that the soybean cyst nematode, a nematode with a similar pathogenic strategy to *M. incognita*, penetrates the roots of the target plant within a day of being inoculated as a juvenile (Lauritis et al., 1983). Our results, of upregulated *GhOPR3*, fall within that timeline and make it likely that this is when the plant defenses will be triggered by the nematode.

If JA was induced by the bacteria in this assay then both *GhLOX1* and *GhOPR3* would have been upregulated upon stimulation by the *Bacillus* spp. rather than just *GhLOX1*. The upregulation of only *GhLOX1* by the *Bacillus* spp. suggests an intermediate jasmonate is responsible for the local and temporal resistance response stimulated by the bacteria. Gleason et al. (2016) determined that cis-(+)-12-oxo-phytodienoic acid (OPDA) was most likely responsible for defense against *M. hapla* in *Arabidopsis*, rather than JA, and may be more important than JA in defense against nematodes. Other studies also implied that OPDA, rather than another intermediate molecule, is important in defense (Stintzi et al., 2001; Bosch et al., 2014; Varsani et al., 2019).

In contrast, *β-1,3-glucanase* was upregulated steadily by the *Bacillus* spp. until there was an almost 2-fold increase in expression stimulated by both *Bacillus* spp. It’s hypothesized that if the experiment was continued for a longer time, this upregulation may increase to larger than 2-fold. The slight increase in *β-1,3- glucanase* expression could indicate an increased activity level of SA as a late term defense response (Li et al., 2003; Zhang et al., 2011). The nematode also upregulated expression of this gene at 24 hours thought to be a result of the plant natural response to the nematode (Hoysted et al., 2017). This would be similar to the upregulation of *GhOPR3* by the nematode after 24 hours. This dynamic between SA and JA after stimulation with a biological control agent has not previously been documented. One of the few other studies that looks at both SA and JA after stimulation with a biological control agent, *Trichoderma* spp. specifically saw a very different interaction between the hormones in tomatoes (Martinez-Medina et al., 2017). In this study, SA was initially stimulated while genes related to JA were only upregulated after about 7 days. These results were very different from our own study which saw no JA response after *Bacillus* spp. stimulation and a long-term response of SA. Another study by Garcia-Gutierrez et al., 2013, observed both SA and JA dependent defenses stimulated by *B. subtilis* UMAF6639 in melon at approximately the same time, which is also very different than our results. Ghahremani et al., 2020 found in tomato *B. firmus* I-1582 induces both JA and SA pathway up-regulation in plants inoculated with the bacteria and nematode in comparison to non-inoculated plants. Many of these studies have not been repeated using the same strain of bacteria on other crops or pathogens. Though systemic resistance by a bacterium can be seen in many different crops against a variety of pathogens, the results are highly variable. Further studies are needed to fully understand indirect control strategies of biological control agents, especially regarding their interactions with the plant and pathogen, in order to successfully implement them in a management strategy.

Our results, in cotton, indicate two *Bacillus* spp. are successful control options that work *via* systemic resistance to manage *M. incognita*. As mentioned previously, it is important that a biological control agent is fully understood to successfully integrate it into an integrated management strategy. Neither species activates JA; rather they activate an intermediate defense molecule, thought to be OPDA, and a potential long term SA response within the plant. While our results identified a mechanism of action of these bacteria, field studies are required before trying to implement any biological control agent as a successful control strategy for a nematode in a commercial setting.

## Conclusion

*Bacillus amyloliquefaciens* QST713 and *B. firmus* I-1582 can cause systemic resistance, as indicated by the split root assay and confirmed in the RT-qPCR. Both of these bacteria increased signaling of an intermediate jasmonate, most likely OPDA, for a short-term defense response and slightly increased SA activity for a long-term defense response. *Bacillus firmus I-1582* may even have two mechanisms of action by which it manages the nematode including the release of metabolites and systemic resistance. Our results also indicate that the techniques used, an *in vitro* assay, a split root assay, and RT-qPCR, can successfully determine systemic resistance. We can also conclude that these two biological control agents are successful in systemically managing *M. incognita*.

## Data availability statement

The original contributions presented in the study are included in the article/supplementary material. Further inquiries can be directed to the corresponding author.

## Author contributions

KG and KL designed the nematode experiments. SP selected the primers. KG preformed the experiments and the data collections. KG ran all the PRC and QPCR with guidance from SP. KG and KL wrote the final version of the manuscript with revisions from SP. All authors contributed to the article and approved the submitted version.
